# Systematic review and meta-analysis: influence of smoking cessation on incidence of pneumonia in HIV

**DOI:** 10.1186/1741-7015-11-15

**Published:** 2013-01-22

**Authors:** Preeti De, Amanda Farley, Nicola Lindson, Paul Aveyard

**Affiliations:** 1 Primary Care Clinical Sciences, University of Birmingham, Birmingham, B15 2TT, UK; 2 Primary Care Health Sciences, University of Oxford, Radcliffe Observatory Quarter, Woodstock Road, Oxford, OX2 6GG, UK

**Keywords:** HIV, meta-analysis, pneumonia, smoking, smoking cessation

## Abstract

**Background:**

Smoking is common in people infected with HIV but cessation support is not a routine part of clinical care. The aim was to assess whether smoking is a risk factor for pneumonia in people with HIV and whether smoking cessation ameliorates excess risk.

**Methods:**

We performed MEDLINE and Embase database searches and included cohort or case-control studies conducted in adult patients infected with HIV extracting a hazard ratio (HR) or odds ratio (OR) that compared the incidence of bacterial pneumonia or pneumonia caused by *Pneumocystis jiroveci *(PCP) between two smoking categories. Studies were appraised for quality and combined using inverse variance meta-analysis.

**Results:**

Fourteen cohort and case-control studies were included. Assessment of outcome was good, but assessment of exposure status was poor. Current smokers were at higher risk of bacterial pneumonia than former smokers: HR 1.37 (95% confidence interval (CI): 1.06, 1.78). There was no evidence that former smokers were at higher risk than never smokers: HR 1.24 (95%CI: 0.96, 1.60). Current smokers were at higher risk of bacterial pneumonia than current non-smokers: HR of 1.73 (95%CI: 1.44, 2.06). There was no evidence that smoking increased the incidence of PCP. The HR for current versus non-smokers was 0.94 (95%CI: 0.79, 1.12), but from case-control studies the OR was 1.76 (95%CI: 1.25, 2.48) with heterogeneity. Confined to higher quality studies, the OR was 0.97 (95%CI: 0.81, 1.16). Residual confounding is possible, but available data suggest this is not an adequate explanation.

**Conclusions:**

Smoking is a risk factor for bacterial pneumonia but not PCP and smoking cessation reduces this risk.

See related article: http://www.biomedcentral.com/1741-7015/11/16

## Background

Life expectancy in patients with HIV has improved dramatically in the era of highly active antiretroviral treatments (HAART), which can prevent the decline in CD4 lymphocyte count and AIDS related morbidity and mortality [1]. However, persons with HIV still face substantially increased health risks compared with the general population. Another threat to health is that the prevalence of cigarette smoking is high amongst people with HIV at around 50% to 70% [2]. Given that many people smoke due to nicotine dependence rather than a desire to smoke and that highly cost-effective treatments for nicotine dependence are available [3], offering smoking cessation treatment to patients with HIV could be an important component of routine clinical care. Currently, smoking cessation support is not routine [2]. The case for offering cessation treatment as part of HIV care rests upon evidence that smoking cessation leads to improved outcomes specific to this population.

Smoking is a risk factor for bacterial pneumonia in the absence of HIV and without evidence of chronic obstructive pulmonary disease. However, it is unclear whether the elevated risk due to smoking is ameliorated by smoking cessation [4,5]. Many people in the general population who develop pneumonia do so without pre-existing conditions and therefore do not receive regular medical assessment and treatment. However, people with HIV undergo regular review with the prime aim of preventing opportunistic infection. Pneumonia is an important cause of morbidity and mortality in patients with HIV, and is one of the most frequent AIDS defining illnesses [6]. If smoking constitutes a risk factor for pneumonia among patients with HIV and if smoking cessation ameliorates that risk, then active management of smoking in people with HIV is supported. The best evidence of causality that smoking cessation ameliorates risk would come from randomized trials of smoking cessation support versus usual care where the outcomes included incidence of pneumonia. We identified no such trials. We therefore conducted a systematic review and meta-analysis of cohort and case-control studies to assess whether smoking cessation reduces the incidence of both bacterial pneumonia and *Pneumocystis jiroveci *pneumonia (PCP) in patients with HIV infection, and looked for evidence of a dose-response relationship to support causality. Furthermore, we examined whether there was evidence that any risk from smoking or benefit from cessation was modified by the use of HAART or by CD4 count.

## Methods

### Eligibility criteria

We included cohort or case-control studies conducted in adult patients infected with HIV virus. The studies had to have sufficient information to extract a hazard ratio (HR) or odds ratio (OR) with 95% confidence intervals (CI) that compared the risk of bacterial pneumonia or PCP incidence between two smoking exposure categories. (HRs require studies to have conducted follow-up and thus are available only from cohort studies. Case-control studies can calculate ORs, which approximate the HR when the event in question is relatively uncommon.)

### Data sources and searches

We searched MEDLINE (1950 to January 2010) and Embase (1947 to January 2010) using a combination of text word and exploded medical subject headings terms related to smoking, HIV and pneumonia using the Ovid interface (Appendix 1 in Additional file 1). Two authors independently searched through the titles and abstracts to identify relevant papers for inclusion, with differences resolved through discussion. We also searched the reference lists and conducted a citation search of included studies. Many studies we included were not concerned with the association between smoking status and incidence of pneumonia but presented these data incidentally, for example as part of a multivariable adjustment for the exposure of interest. We therefore searched the full texts of all studies with relevant outcomes even if there was no mention of smoking in the title or abstract.

### Data extraction and quality assessment

Two reviewers independently extracted data and resolved disagreements by recourse to the papers. We extracted data on demographic and clinical characteristics of the population (age, gender, use of HAART, CD4+ count) and on the methods of assessing exposure and outcome measures. We assessed whether the effect estimate was adjusted for the effects of potential confounders: use of intravenous street drugs, alcohol consumption, socio-economic status (such as educational attainment, income), CD4 count, viral load, and use of HAART. Each of these factors is associated with the incidence of pneumonia and could plausibly be associated with smoking.

We assessed study quality based on methods outlined by Altman [7]. We awarded points for assessment of exposure (smoking), outcome (pneumonia) and whether potential confounders were either balanced between exposure groups or controlled for in the analysis (Appendix 2 in Additional file 1). In many developed countries, 2% to 3% of smokers stop smoking annually, therefore ongoing assessment of current smoking status would be needed to ensure that people defined as current smokers were actually exposed. More importantly, almost all people who have recently stopped relapse to smoking [8], so we awarded points for baseline assessment that defined former smokers as not having smoked for at least a year. For the assessment of outcome, our ideal definition included each of the following: compatible physical symptoms, microbiological confirmation, and radiographic confirmation. As presented in some studies, we included either response to antibiotics or microbiological confirmation as alternative confirmatory signs. Additional points were awarded if outcome assessors were blinded to exposure status. The maximum point score was 16 and we classified studies as high quality when they scored six or more, which was the median score. We considered and decided against the use of sensitivity analysis based on key characteristics, namely the quality of exposure assessment and outcome assessment. Studies varied little in these two aspects and so the scoring system mainly reflected differences in the number of confounders controlled.

### Data synthesis and analysis

Risk estimates were extracted as adjusted HRs or ORs with 95%CIs. If the risk estimates or 95%CIs were not presented, we calculated them from presented data using methods described by Parmar *et al. *[9], or using OpenEpi software [10]. Where risk estimates were presented as current or former smokers versus never smokers, we compared current smokers with former smokers using indirect comparison [11].

Adjusted ORs or HRs (where available) were combined separately from case-control and cohort studies, calculating summary ORs or HRs respectively using inverse variance fixed effects methods implemented in Review Manager 5.0.24. We assessed heterogeneity using the I^2 ^statistic and publication bias using funnel plots (shown in Additional file 1). It is general convention to view I^2 ^values of less than 25% as showing no appreciable heterogeneity, between 25% and 50% as moderate, and more than 50% as substantial heterogeneity. In sensitivity analysis, we confined our analysis to studies defined as high quality. We combined studies in subgroups where participants did not use HAART or where HAART was used and assessed whether use of HAART modified the association between smoking and pneumonia by the χ^2 ^test for heterogeneity. We assessed whether the association between smoking and pneumonia incidence was modified by CD4 count. We used within-study data for this, reporting risk estimates by CD4 strata.

## Results

**Figure 1:**
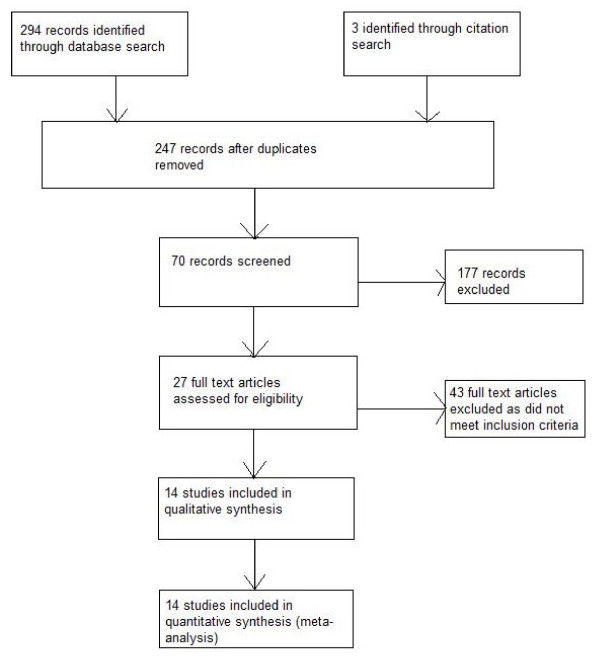
**Flow diagram**.

Our search identified 294 studies, of which we obtained 27 full-text articles and 14 studies were included in the review (Figure 1). Of the 14 observational studies included, 12 examined the effect of smoking status on the incidence of bacterial pneumonia, with seven (58%) including patients treated with HAART, and five examined the effect of smoking status on the incidence of PCP, with two (40%) including patients treated with HAART (Table 1). There were five case-control studies and nine cohort studies. In 10 studies the populations were based in the USA, three were based in Europe, and one in South Africa. The prevalence of smoking at cohort inception ranged from 39% to 92% with a median of 60%. Most patients were men (range 56% to 99%, median 77%) and the average age of the population was about 40 years (Table 1). Average length of follow-up in the cohort studies ranged from 1.3 to 10 years (median 4 years), a total of about 62,000 person-years of follow-up in the cohort studies. There were 1,409 participants included in the case-control studies.

**Table 1:** Characteristics of participants in included studies for bacterial pneumonia and *Pneumocystis jiroveci *pneumonia.

Reference	Number. of participants	Men (%)	Mean/median age(SD) in years	Smokers (%)	Non-smokers (%)	Mean/median CD4+ count	Use of HAART
**Bacterial pneumonia**							

[12]	40	75	Median 30 to 40	NR	NR	NR	Pre HAART era
[21]	867	99	Mean 50 (10)	63%	22% former smokers,15% never smokers	NR	Post-HAART era
[13]	509	100	Median 30	80%	20% non-smokers	177 cells/μL	Post-HAART era
[23]	5472	73	Median 43	41%	25% former, 34% never smokers	>350 cells/μL	Post-HAART era
[14]	1130	NR	NR	NR	NR	NR	Pre-HAART era
[15]	885	0 (all female)	Mean 36	76%	24%	NR	Pre and Post HAART era
[16]	1203	77	Mean 36	66% unknown orcurrent smokers	34% currently not smoking	279 cells/μL	Post-HAART era
[17]	300	56	Mean 37 (7)	92%	8% non-smokers	327 cells/μL	Post-HAART era
[18]	285	NR	Mean 34 (8)	55%	45% non-smokers	182 cells/μL	Pre-HAART era

**Bacterial pneumonia and *Pneumocystis jiroveci *pneumonia**							

19]	3221	83	Mean 37	57%	19% former smokers,24%, never smoker	327 cells/μL	Pre-HAART era
[20]	232	100	Median 36 to 38	46%	54%	NR	Pre-HAART era
[25]	521	58	Mean 42 (9)	63%	12% former smokers,25% never smokers	172 cells/μL	Post-HAART era

***Pneumocystis jiroveci *pneumonia only**							

[22]	2499	100	NR	39%	61%	>200 cells/μL	Pre-HAART era
[24]	54	59	Mean 40	NR	NR	257 cells/μL	Post-HAART era

HAART: highly active antiretroviral treatments; NA: not applicable; NR: not reported.

### Quality assessment

Seven of the 14 studies were not primarily concerned with the association between smoking and the incidence of pneumonia, and details of the definition of smoking exposure were scant [12-18]. Even in studies focused on smoking, the definitions of smoking exposure given were suboptimal and studies lost quality points as a consequence [19-25]. No studies dealt with the fact that former smokers might relapse while current smokers might become former smokers. Most studies met the quality criteria for outcome assessment. Eight of the 12 estimates for bacterial pneumonia risk were adjusted for some predefined confounders [12,14-16,19,21,23,25], with individual studies adjusting for between three and seven confounders (median four). Four of the five studies on PCP adjusted for confounding, range one to four, median 2/3 [19,22,24,25]. We classified six studies scoring six and above as high quality, of which five had outcomes of bacterial pneumonia [12,14-16,23] and one of PCP [22] (Table 2).

**Table 2:** Methodological characteristics of studies.

Author (year)	Study design	Exposure assessment			Outcome assessment		Number of confounders controlled	Study quality score
				
		Definition of smoking	Definition of non smoking	Method of assessingsmoking status	Definition ofpneumonia	Blinding ofassessors toexposure status (cohort studies)		
**Bacterial pneumonia**								

[12]	Case control	>1 cig/day	Never smoked	Medical history	2	0	3	7
[21]	Cohort	NR	Former and never, otherwise undefined	Questionnaire or clinical notes	1	0	4	5
[13]	Nested case-control	NR	NR	NR	2	1	0	3
[23]	Cohort	NR	NR	Interview	2	1	4	7
[14]	Cohort	NR	Never smokers <100 cigarettes in lifetime	NR	2	0	4	6
[15]	Cohort	NR	Not current smokers	NR	2	0	7	9
[16]	Cohort	NR	NR	Questionnaire	2	0	4	6
[17]	Cohort	NR	NR	Questionnaire	2	0	0	2
[18]	Case control	>6 cigs/day	NR	NR	2	0	0	3

**Bacterial pneumonia and *Pneumocystis jiroveci *pneumonia**								

[19]	Cohort	NR	Self-defined	Clinical notes	0	0	4	4
[20]	Cohort	Cigarettes/day in multiple categories	NR	Questionnaire	1	0	0	2
[25]	Case control	NR	NR	Questionnaire	2	0	3	5

**Pneumocystis *jiroveci *pneumonia only**								

[22]	Cohort	Defined according to daily cigarette consumption and change in smoking status over time	Not currently smoking	Questionnaire	2	0	2	6
[24]	Case control	Current tobacco use	Current non-smoking	Questionnaire and clinical notes	2	0	1	4

NR: not reported.

### Association between smoking cessation and incidence of bacterial pneumonia

**Figure 2:**
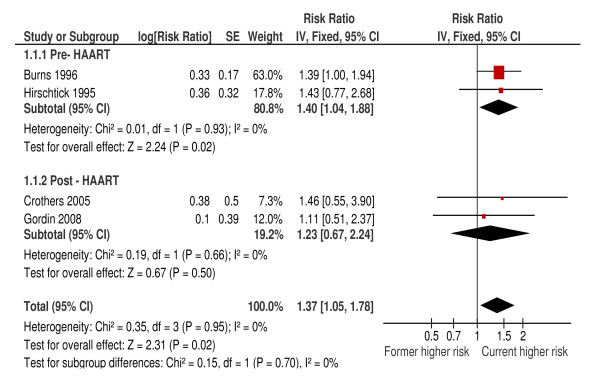
**Comparison of risk of bacterial pneumonia between HIV seropositive current smokers and former smokers**.

One cohort study provided an HR for the difference in incidence between current and former smokers [19]. We used indirect methods to calculate HRs for this comparison from data in three studies [14,21,23]. In comparison to former smokers, current smoking was associated with a 37% increased risk in developing bacterial pneumonia (HR 1.37; 95%CI: 1.06, 1.78; *P *= 0.02) (Figure 2). Effect estimates were similar in all four studies with an I^2 ^value of 0%, with no evidence of difference in the pre- to post-HAART eras *(P = *0.70). After excluding studies of lower quality, the summary estimate did not change greatly, but was no longer significant (HR 1.28; 95%CI: 0.93, 1.76).

### Former smokers versus never smokers and incidence of bacterial pneumonia

**Figure 3:**
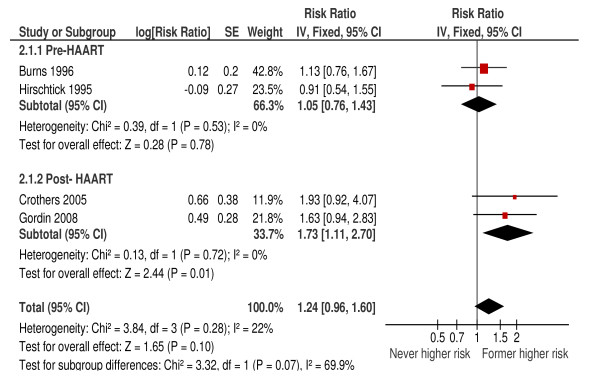
**Risk of bacterial pneumonia in former versus never smokers (cohort studies)**.

There was no evidence that former smokers were at greater risk of bacterial pneumonia than never smokers (HR 1.24; 95%CI: 0.96, 1.60; *P *= 0.10; I^2 ^= 22%) (Figure 3), which remained the case after exclusion of lower quality studies (HR 1.29; 95%CI: 0.79, 2.10). Subgroup analysis for treatment era showed some evidence that former smokers on HAART were at higher relative risk from their past smoking than those not on HAART although the difference between groups was not significant (*P *= 0.07).

### Current smokers versus current non-smokers and incidence of bacterial pneumonia

**Figure 4:**
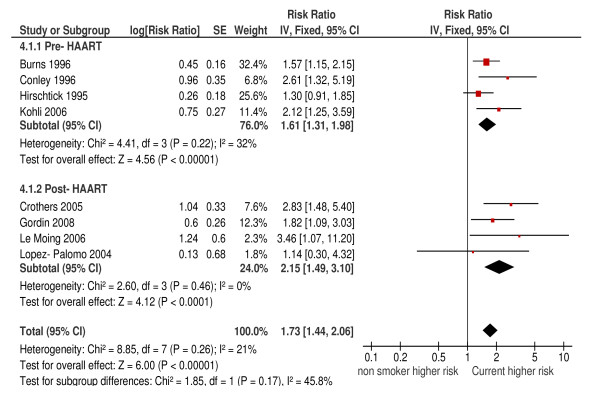
**Risk of bacterial pneumonia in current versus non-smokers (cohort studies)**.

**Figure 5:**
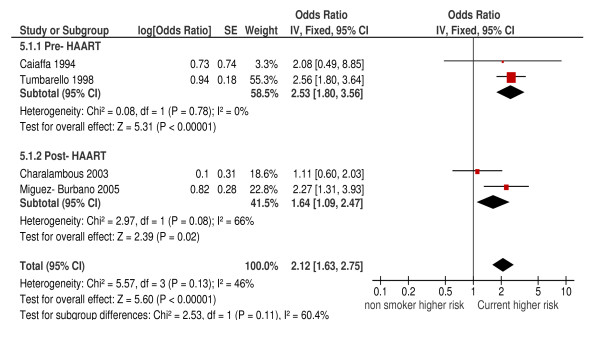
**Risk of bacterial pneumonia in current versus non-smokers (case-control studies)**.

These results, which give direct evidence on the possible effect of cessation, were supported by more extensive indirectly relevant data. We pooled eight cohort studies where the incidence of pneumonia in current smokers was compared with a combined group of never and former smokers at study entry (hereafter referred to as non-smokers for simplicity). This produced a summary HR of 1.73 (95%CI: 1.44, 2.06; *P *<0.001; I^2 ^= 21%) (Figure 4). Confined to four higher quality cohort studies, the HR was 1.64 (95%CI: 1.28, 2.10; I^2 ^= 30%). There were four case-control studies comparing risk of pneumonia in current and non-smokers, which gave a pooled OR of 2.12 (95%CI: 1.63, 2.75; *P *<0.001) but with more substantial heterogeneity (I^2 ^= 46%) (Figure 5). No case-control studies were rated high quality. In both these meta-analyses there was weak evidence that the association between smoking and bacterial pneumonia was modified by the use of HAART. For current versus non-smokers, the p value for subgroup differences for cohort studies was 0.17 (Figure 4) and for case-control studies it was 0.11 (Figure 5). However the direction of possible effect modification differed, with participants using HAART who smoked being at higher risk from smoking in cohort studies and at lower risk in case-control studies.

### The association between smoking cessation and PCP

Three of five studies containing data on PCP were cohort studies [19,20,22]. One study reported data that allowed us to calculate the risk of PCP for current compared with former smokers [19]. There was no evidence that the risk differed (relative risk (RR) 0.81; 95%CI: 0.55, 1.20).

**Figure 6:**
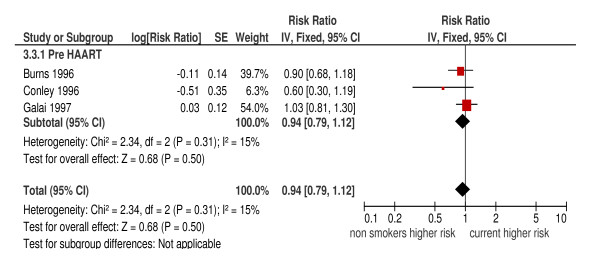
**Risk of *Pneumocystis jiroveci *pneumonia in current smokers compared to non-smokers (cohort studies)**.

**Figure 7:**
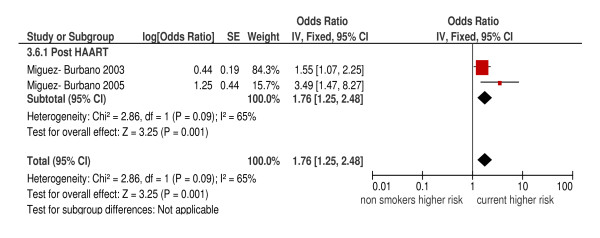
**Risk of *Pneumocystis jiroveci *pneumonia in current smokers compared to non-smokers (case-control studies)**.

The overall pooled estimate for current versus non-smokers from the three cohort studies gave an HR of 0.94 (95%CI: 0.79, 1.12; *P *= 0.31; I^2 ^= 15%), and from the two case-control studies gave an OR of 1.76 (95%CI: 1.25, 2.48; *P *= 0.001) but with substantial heterogeneity (I^2 ^= 65%) (Figures 6 and 7). The three cohort studies were all conducted in the pre-HAART era and the two case-control studies in the post-HAART era. For the two higher quality cohort studies, the summary HR was 0.97 (95%CI: 0.81, 1.16) with no heterogeneity (I^2 ^= 0%). There were no case-control studies with a quality score six or above.

### Assessing the effect of confounding factors

Four studies reported data on both unadjusted and adjusted HRs in bacterial pneumonia. Hirschtick *et al*. reported that adjustment reduced the HR between current and never smokers but direct numerical comparisons were not possible [14]. In Gordin *et al*., adjustment for confounding by intravenous drug use, use of HAART, CD4 count and viral load reduced the HR slightly, from 1.80 to 1.64 (former versus never) and 2.19 to 1.82 (current versus never) [23]. In Caiaffa *et al*., adjustment for CD4 count, age and illicit drug use increased the OR slightly from 2.00 to 2.08 [12]. In Miguez-Burbano *et al*., adjustment for CD4 count, viral load and use of HAART changed the OR from 1.80 to 2.28 [25].

There was only one study that allowed comparison between unadjusted and adjusted estimates of the risk of PCP. Miguez-Burbano *et al*. reported that the OR for current versus non-smokers decreased on adjustment from approximately 16 to 1.56 [24].

### Dose-response relationships between smoking and outcomes

No studies examined the effect of years since cessation on the incidence of pneumonia or even reported on years of cessation achieved in former smokers. Conley *et al*. reported a significant linear trend for risk of bacterial pneumonia with heaviness of consumption in current smokers, but no trend for PCP [20]. Miguez-Burbano *et al*. reported a significant dose-response relationship, with the odds of bacterial pneumonia increasing by 3% per cigarette per day in a linear trend analysis [25]. Galai *et al*. reported no association between number of cigarettes smoked and increasing risk of PCP [22].

### Is the association between smoking and bacterial pneumonia or PCP modified by CD4 count?

Three studies reported data for bacterial pneumonia, but none allowed a quantitative summary of the effect estimates of smoking on pneumonia by CD4 count category. Hirschtick *et al*. reported that a low CD4 count (<200 lymphocytes per microliter) was associated with an increased relative hazard from current smoking [14], Miguez-Burbano *et al*. reported a lower relative hazard [25], and Burns reported no difference in relative hazard by CD4 count [19]. Galai *et al*. reported that CD4 count did not significantly modify the effect of smoking on incidence of PCP [22].

## Discussion

We found strong statistical evidence that, in patients with HIV, current smoking was associated with an approximate 70% to 100% increased risk of bacterial pneumonia compared with non-smokers, and moderate evidence that stopping smoking decreased this by about 27%. There was some evidence that former smokers were at slightly increased risk compared with never smokers, which was not significant, however the estimate was insufficiently precise to exclude a substantial increased risk. There was no good evidence that the risk of smoking was modified either by the use of HAART or by CD4 count. There was mixed evidence that smoking increased the risk of PCP, but higher quality evidence indicated no substantial increase in risk of PCP from current smoking. The evidence from all studies was somewhat clouded by poor definition of exposure status (smoking) and failure to control for a full range of confounders. However, studies that reported the effects of adjustment suggested that adjustment for several confounders had an insubstantial effect on these estimates.

The strengths of this study relate to the comprehensive search of studies that had relevant outcomes even though the titles or abstracts included no mention of smoking. We found seven studies this way that did not have an investigation of smoking as a risk factor for pneumonia as their prime aim. It is plausible that several cohort studies investigating smoking as a risk factor failed to find a significant association, and therefore did not publish data. Incorporating studies where smoking was not the main focus offers reassurance that the association is not due to publication bias and funnel plots also suggested no evidence of bias.

There are some limitations with the review and the data we reviewed. We might have searched a larger number of databases and we did not search the grey literature. We included observational data, and there were inherent limitations. First, the measurement of smoking was suboptimal. Over time, people who were smokers at baseline would have stopped smoking, while some ex-smokers might have relapsed; this will mix exposure assignment and generally underestimate any true risk of smoking on pneumonia [26]. Another limitation is that few studies adjusted for a full range of confounders. People who smoke are more likely than people who do not to have other factors associated with an increased risk of pneumonia, for example, intravenous drug use or failure to adhere to HAART. Our ability to assess the importance of this explanation was compromised by failure to report unadjusted and adjusted estimates, but those that were presented suggested a small effect of adjustment and this is unlikely to explain the associations observed.

Taken together, we believe this systematic review gives reasonable evidence that the association between smoking and bacterial pneumonia is causal in HIV patients. There are no strong reasons to doubt the validity of the association and there was a dose-response relationship between smoking and risk of pneumonia. There are supporting data that suggest biologically plausible mechanisms, such as reduced local defenses in the lung. Smokers with HIV have lower concentrations of CD4 and CD8 lymphocytes in lung tissue and lower concentrations of cytokines IL-1β and TNF-α [27]. There is also evidence that smoking reduces the phagocytic function of alveolar macrophages in individuals infected with HIV [28]. Another possible explanation is that free radicals in tobacco smoke increase oxidative stress within cells and therefore enhance the likelihood of infection [29].

This review produced inconclusive evidence on whether there is an association between current smoking and risk of PCP in patients with HIV. There was no evidence of a dose-response relationship and, given the weight of evidence, we conclude that smoking is not a risk factor for development of PCP. HAART prevents the occurrence of PCP and antibiotic prophylaxis against PCP can be stopped in people with a CD4 count greater than 100 cells/μL [30]. It is clear then that CD4 count is critical and this reinforces our conclusion that smoking is not an important risk factor for this pneumonia.

In this meta-analysis, the relative risk of bacterial pneumonia in current smokers compared to non-smokers was 26% higher than the relative risk of current smokers compared to non-smokers. In western countries, where nearly all of these studies were completed, most non-smokers aged around 40 years (the average age of participants in included studies) are never smokers rather than former smokers [31]. The risk of former smokers in comparison to never smokers was imprecisely estimated and we could not therefore exclude a continued risk in former smokers. These data are clouded further by the definitions of smoking status used in these studies. We cannot therefore be clear that the risk of pneumonia for former smokers is the same as never smokers, but it is clear that it is lower than among current smokers.

To our knowledge, no other systematic reviews have been published on this topic, but narrative reviews have addressed it [32-34]. This is the first review to explicitly compare current smokers with former smokers. All but one study published data comparing the risk of current smoking to never smokers, but a person who smokes cannot become a never smoker. Using indirect comparisons allowed us to estimate the association that is most clinically relevant. We were, however, unable to estimate how long the risk of current smoking remains elevated after stopping before the benefits of cessation are manifest. There are numerous cohort studies of people with HIV and they are likely to have recorded data on smoking repeatedly during the follow-up of the cohorts. It would therefore be useful for these cohorts to examine the difference in risk by time since cessation and including these data would allay concerns that smoking status is likely to have changed over time. There is evidence that smoking predisposes to tuberculosis, but rather less data on the effect of smoking cessation on tuberculosis [35]. Such associations could also be investigated in these studies.

These results have direct clinical implications. The prevalence of smoking was high in these cohorts, much higher than the population in general, in keeping with the higher prevalence in gay men and intravenous drug users [36,37]. Two studies reported incidence rates for bacterial pneumonia, the weighted mean of which was 7.8 per 100 person-years in smokers [14,15]. This means that 25% of smokers would develop pneumonia over 10 years due to their smoking that would have been prevented by smoking cessation. Of cases of pneumonia in people with HIV, 22% are due to current smoking if the median prevalence of smoking in these studies (60%) applies generally. Thus it seems imperative that physicians caring for people with HIV become proficient in smoking cessation treatment, regularly offer treatment, and support their patients to stop smoking. Brief advice to stop smoking is insufficient. The large majority of people will fail to stop with advice alone [38]. Many smokers who are gay or current or former intravenous drug users want to stop smoking but find this difficult due to nicotine addiction; effective treatment programs exist that can ameliorate this [3]. The decision on whether such programs become routine practice in HIV medicine depends upon showing that they are cost-effective in this population. Smoking cessation programs have been called 'among the most cost-effective of all healthcare interventions' (p7 of [39]) in the general population, so it seems likely that they would be similarly cost-effective in people with HIV. There are several small scale trials published of treatment for smoking cessation in people with HIV [40-43], though there is no reason to imagine that treatment regimens in this subgroup need be very different from standard smoking cessation treatment. However, it was outside the scope of this review to examine evidence of efficacy in supporting cessation or its cost-effectiveness.

## Conclusions

There is reasonable evidence that smoking cessation is casually associated with a reduced risk of bacterial pneumonia but not PCP in people with HIV. Smoking cessation programs should be incorporated into HIV treatment programs.

## Abbreviations

CI: confidence interval; HAART: highly active antiretroviral treatments; HIV: human immunodeficiency virus; HR: hazard ratio; IL: interleukin; OR: odds ratio; PCP: *Pneumocystis jiroveci *pneumonia; TNF: tumor necrosis factor.

## Competing interests

PA has done research and consultancy for manufacturers of smoking cessation medication. All other authors declare that they have no competing interests.

## Authors' contributions

AF conceived the study and all authors contributed to developing the protocol. All authors searched the literature. PD and PA extracted and analyzed the data. PD and PA wrote the first draft and all authors edited it. All authors have read and approved the final manuscript.

## Pre-publication history

The pre-publication history for this paper can be accessed here:

http://www.biomedcentral.com/1741-7015/11/15/prepub

## Supplementary Material

Additional file 1 **Search strategy, quality criteria, and funnel plots**. A line-by-line search strategy, the criteria used to assign quality points to studies, and the funnel plots of each of Figures 2 to 7. Click here for file
