# Supplementary material for: Systematic review and meta-analysis: influence of smoking cessation on incidence of pneumonia in HIV
Source: BMC Med. 2013 Jan 22;11:15. doi: 10.1186/1741-7015-11-15 (PMC3606464; doi:10.1186/1741-7015-11-15)
Supplement: Additional file 1 — Search strategy, quality criteria, and funnel plots. A line-by-line search strategy, the criteria used to assign quality points to studies, and the funnel plots of each of Figures 2 to 7. [file 1741-7015-11-15-S1.DOC]

**Appendix 1 Search strategy**

Exp smoking/

Exp smoking cessation/

Exp tobacco/

(smoking or smoke or smoker$ or tobacco or cigarette$).mp.

1 or 2 or 3 or 4

Exp HIV -1/ or human immunodeficiency virus 1/

(HIV or human immunodeficiency virus or human immune deficiency virus or HIV infectio$ or human immune deficiency virus infection).mp.

Exp AIDS / or acquired immunodeficiency syndrome/ or acquired immune deficiency syndrome/

6 or 7 or 8

5 and 9

(Opportunistic infections or opportunistic infectio$ or AIDS related opportunistic infection$).mp.

Exp Pneumonia/ or bronchopneumonia/ or pneumonia, bacterial/ or pneumonia, pneumocystis/ pneumocystis carinii pneumonia/ or community acquired pneumonia/

11 or 12

10 and 13

“review”

15 not 16

Remove duplicates from 17

**Appendix 2 Coding criteria for study quality assessment**

**Exposure assessment**

Clear definition of smoking status at baseline

0 points - no classification

1 point – definition of current smoker, such as currently smoking more than one cigarette per day

2 points- definition of current smoker as well as former smoker as not having smoked for a year previous to the start of study and definition of never smoker as having smoked < 100 cigarettes in their lifetime

Continuous assessment of smoking

0 points – no classification

1 point – annual report (which was adjusted for as a time varying covariate)

2 points – semi annual report (which was adjusted for as a time varying covariate)

Blinding of assessors to outcome status when assessing exposure in case-control studies

0 points – no blinding

1 point – blinding of assessors

**Assessing major biases- loss to follow up in cohort studies and selection of controls for case- control studies**

For cohort studies,

0 points – loss to follow up unequal in comparison arms

1 point – loss to follow up > or equal to 30% equal in comparison arms

2 points – loss to follow up < 30% and equal in comparison arms

For case control studies

0 points –controls selected from hospital setting

2 points – community controls selected

**Outcome assessment**

Blinding of assessors to exposure status for cohort studies

0 points – no blinding

1 point – blinding of assessors

Clear definition of pneumonia

0 points - no definition

1 point – verified by some classification system falling short of the one below

2 points – combination of clinical and radiographic confirmation with either response to treatment or microbiological examination

**Confounders**

One point was awarded for each confounder adjusted for from the list:

IV drug use

Use of HAART

CD4 cell count

Viral load

Alcohol consumption

Socioeconomic class

Education status

**Appendix 3a. Funnel plot to assess level of publication bias for comparison of risk of bacterial pneumonia between HIV seropositive current smokers and former smokers**

**3b. Funnel plot to assess level of publication bias for comparison of risk of bacterial pneumonia between HIV seropositive former smokers and never smokers**

**3c. Funnel plot to assess level of publication bias for comparison of risk of bacterial pneumonia between HIV seropositive current smokers and non smokers (cohort studies)**

**3d. Funnel plot to assess level of publication bias for comparison of risk of bacterial pneumonia between HIV seropositive current smokers and non smokers (case control studies)**

**3e. Funnel plot to assess level of publication bias for comparison of risk of PJP between HIV seropositive current smokers and non smokers (cohort studies)**

**3f. Funnel plot to assess level of publication bias for comparison of risk of PJP between HIV seropositive current smokers and non smokers (case control studies)**
